# SPG15 protein deficits are at the crossroads between lysosomal abnormalities, altered lipid metabolism and synaptic dysfunction

**DOI:** 10.1093/hmg/ddac063

**Published:** 2022-03-21

**Authors:** Lara Marrone, Paolo M Marchi, Christopher P Webster, Raffaele Marroccella, Ian Coldicott, Steven Reynolds, João Alves-Cruzeiro, Zih-Liang Yang, Adrian Higginbottom, Mukhran Khundadze, Pamela J Shaw, Christian A Hübner, Matthew R Livesey, Mimoun Azzouz

**Affiliations:** Sheffield Institute for Translational Neuroscience (SITraN), Department of Neuroscience, University of Sheffield, Sheffield, UK; Department of Neuroscience, Janssen Pharmaceutica, Beerse, Belgium; Sheffield Institute for Translational Neuroscience (SITraN), Department of Neuroscience, University of Sheffield, Sheffield, UK; Sheffield Institute for Translational Neuroscience (SITraN), Department of Neuroscience, University of Sheffield, Sheffield, UK; Sheffield Institute for Translational Neuroscience (SITraN), Department of Neuroscience, University of Sheffield, Sheffield, UK; Sheffield Institute for Translational Neuroscience (SITraN), Department of Neuroscience, University of Sheffield, Sheffield, UK; Academic Unit of Radiology, Department of Infection, Immunity and Cardiovascular Disease, University of Sheffield, Royal Hallamshire Hospital, Sheffield, UK; Sheffield Institute for Translational Neuroscience (SITraN), Department of Neuroscience, University of Sheffield, Sheffield, UK; Sheffield Institute for Translational Neuroscience (SITraN), Department of Neuroscience, University of Sheffield, Sheffield, UK; Sheffield Institute for Translational Neuroscience (SITraN), Department of Neuroscience, University of Sheffield, Sheffield, UK; Institute of Human Genetics, Jena University Hospital, Friedrich-Schiller-University Jena, Jena, Germany; Sheffield Institute for Translational Neuroscience (SITraN), Department of Neuroscience, University of Sheffield, Sheffield, UK; Institute of Human Genetics, Jena University Hospital, Friedrich-Schiller-University Jena, Jena, Germany; Sheffield Institute for Translational Neuroscience (SITraN), Department of Neuroscience, University of Sheffield, Sheffield, UK; Sheffield Institute for Translational Neuroscience (SITraN), Department of Neuroscience, University of Sheffield, Sheffield, UK

## Abstract

Hereditary spastic paraplegia type 15 (HSP15) is a neurodegenerative condition caused by the inability to produce SPG15 protein, which leads to lysosomal swelling. However, the link between lysosomal aberrations and neuronal death is poorly explored. To uncover the functional consequences of lysosomal aberrations in disease pathogenesis, we analyze human dermal fibroblasts from HSP15 patients as well as primary cortical neurons derived from an SPG15 knockout (KO) mouse model. We find that SPG15 protein loss induces defective anterograde transport, impaired neurite outgrowth, axonal swelling and reduced autophagic flux in association with the onset of lysosomal abnormalities. Additionally, we observe lipid accumulation within the lysosomal compartment, suggesting that distortions in cellular lipid homeostasis are intertwined with lysosomal alterations. We further demonstrate that SPG15 KO neurons exhibit synaptic dysfunction, accompanied by augmented vulnerability to glutamate-induced excitotoxicity. Overall, our study establishes an intimate link between lysosomal aberrations, lipid metabolism and electrophysiological impairments, suggesting that lysosomal defects are at the core of multiple neurodegenerative disease processes in HSP15.

## Introduction

Hereditary spastic paraplegias (HSPs) are inherited neurodegenerative disorders characterized by gradual lower limb spasticity. While spasticity can manifest as the only symptom, it can also occur in conjunction with additional neurological signs, such as cognitive decline, visual impairment, cerebellar ataxia and Parkinsonism (1,2), indicating widespread non-motor pathology. This occurs in a minority of HSP cases, which are defined as ‘complex’. To date, there is no therapy capable of delaying or halting HSPs, and treatments provide purely symptomatic relief. Although life expectancy is not significantly shortened, patients live strongly compromised lives, requiring incessant care and assistance. Hence, HSPs represent an important unmet medical need. There are more than 80 subtypes of HSP, each linked to mutations in a different genetic locus (3). HSP type 15 (HSP15) is a complex form of autosomal recessive HSP caused by mutations affecting the *ZFYVE26* gene, encoding the Spastizin/SPG15 protein (4).

HSP15 mutations are generally associated with the occurrence of premature stop codons, which induce the degradation of *SPG15* transcripts by nonsense-mediated decay (5). Consequently, the SPG15 protein fails to be synthesized, and pathology arises as a result of a loss of function. The cellular functions of SPG15 remain poorly defined, though emerging evidence suggests the structural involvement of SPG15 in the recently described AP-5 adaptor protein complex. AP-5 has been implicated in sorting and recycling cargoes from late endosomes and lysosomes (6,7), which most parsimoniously explains why mutations in SPG15 produce aberrations in the endo-lysosomal pathway (8). In addition, loss of SPG15 protein hampers the process of autophagic lysosome reformation that underlies lysosome regeneration from autolysosomes (ALs) during autophagy (9,10), leading to the accumulation of undigested material thought to produce neuronal damage. However, the link between endo-lysosomal aberrations and neuronal death has not been fully elucidated.

In this study, we dissect the functional consequences of SPG15 protein depletion in disease pathogenesis, with a particular focus on the molecular mechanisms linked to endo-lysosomal aberrations. We show that abnormalities in endo-lysosomal vesicles (hereafter collectively referred to as lysosomes) in HSP15 are associated with defective anterograde transport, impaired neurite outgrowth, axonal swelling and reduced autophagic flux. We additionally demonstrate that lysosomal storage pathology culminates in the abnormal retention of lipids within lysosomes. Consistent with lysosomal lipid imbalances generating increased intracellular levels of calcium (11), we determine that SPG15 protein depletion also leads to increased vulnerability to glutamate-mediated excitotoxicity. Further, because the endosomal vesicular pathway, which is disrupted in HSP15, regulates the supply of synaptic vesicles, we examine synaptic properties and find that loss of SPG15 protein is associated with altered glutamatergic synaptic events. Our data lead us to propose a model of cortical dysfunction in HSP15, whereby lysosomal impairment induces increased vulnerability to excitotoxicity that is exacerbated by elevated synaptic glutamatergic transmission.

## Results

### Patient-derived fibroblasts exhibit lysosomal aberrations and increased vulnerability

**Figure 1:**
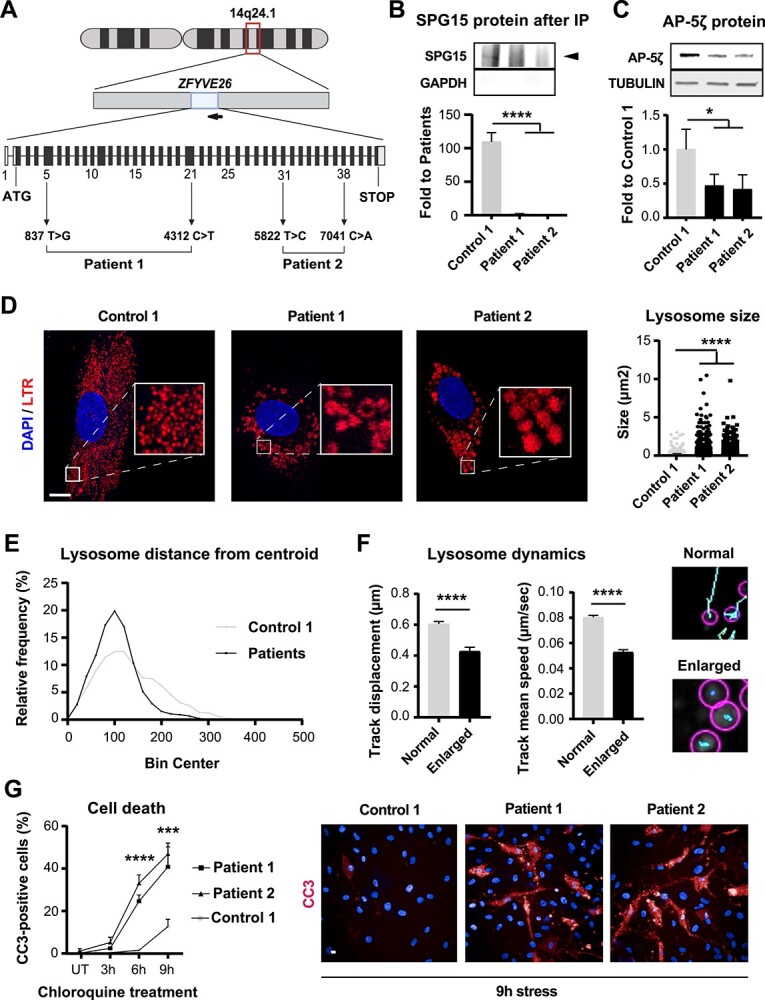
Hallmarks of SPG15 pathology in fibroblasts. (**A**) Schematic of the *ZFYVE26* locus. The gene, consisting of 42 exons (solid boxes), is located on chromosome 14q24.1 and encoded in the reverse DNA strand (arrow pointing left). Mutations affecting the HSP15 patients included in this study are highlighted. (**B**) The SPG15 protein is absent in patient cells. Immunoblotting for SPG15 following immunoprecipitation of endogenous SPG15 protein confirms the presence of an SPG15-specific band only in Control cells (black arrowhead). *N* = 4, Unpaired *t*-test. Error bars represent SD. ^****^ corresponds to *P* < 0.0001. (**C**) Immunoblotting shows a significant reduction in AP-5ζ in patients compared to controls. *N* = 3. Unpaired *t*-test, error bars represent SD. ^*^ corresponds to *P* < 0.05. (**D**) LTR-positive organelles of SPG15 patients are aberrantly enlarged. Confocal micrographs show the endo-lysosomal compartment labeled with Lysotracker Red DND-99 (LTR). Boxed regions are zoomed-in in the insets (left). As confirmed with image quantification (right), patient lysosomes are significantly larger than normal. Scale bar = 10 μm. *N* = 3 (independent experiments, each evaluating at least 100 lysosomes). Mann–Whitney test. ^****^ corresponds to *P* < 0.0001. (**E**) Enlarged lysosomes are located perinuclearly. Quantification of the relative percentage of lysosomes exhibiting varying distances from the cell’s centroid expressed as ‘bin centers’. (**F**) Enlarged lysosomes exhibit impaired dynamics. Quantification of lysosomal track displacement and mean speed from live-cell imaging reveals a significant reduction in the motility of enlarged lysosomes (left). Snapshots from videos showing segmented lysosomes and tracks produced over time provide a visual clue (right). Micrographs display detected lysosomes (pink circles) and their respective tracks (light blue lines). *N* = 3 (independent experiments, each evaluating at least 100 lysosomes). Mann–Whitney test, error bars represent SEM. ^****^ corresponds to *P* < 0.0001. (**G**) SPG15 cells are more vulnerable. A time-course of fibroblast susceptibility to apoptotic cell death over chloroquine treatment for 3, 6 and 9 h shows that patient cells exhibit significantly higher apoptotic rates at 6 and 9 h post-treatment (left) as illustrated by confocal micrographs of cells stained with CC3 (right). *N* = 3 (independent experiments, each evaluating at least 500 cells). Scale bar = 10 μm. Mann–Whitney test, error bars represent SEM. ^***^ and ^****^ correspond to *P* < 0.001 and *P* < 0.0001, respectively.

Our initial *in vitro* studies on HSP15 pathology focused on human dermal fibroblasts. HSP15 patients typically carry premature stop codons that trigger the degradation of *SPG15* transcripts by nonsense-mediated decay (4). Consequently, *SPG15* mRNA levels are drastically reduced and the SPG15 protein remains untranslated. We obtained cells from two HSP15 patients, whose genetic mutations are displayed in Figure 1A. While Patient 1 had two nonsense mutations (Arg1438^*^, Tyr279^*^), Patient 2 exhibited a combination of a nonsense and a missense mutation (Cys2347^*^, Leu1941Pro). We first compared *SPG15* mRNA levels between patient-derived and healthy control fibroblasts, which included two individuals carrying only one mutant allele (Carrier 1 and Carrier 2), as well as a young and an elderly subject carrying the WT SPG15 sequence for both alleles (Control 1 and Control 2, respectively). As expected, RT-qPCR using primers targeting the 5′ end of the *SPG15* transcript revealed that *SPG15* levels were reduced to half in both heterozygous carriers (Supplementary Material, Fig. S1A), while Patient 1 showed the lowest levels of *SPG15* mRNA. Of note, transcript levels of Patient 2 were not significantly different from those of the carriers, which is in line with the presence of a nonsense mutation in only one allele.

We next examined SPG15 protein levels (Supplementary Material, Fig. S1B) and confirmed that SPG15 was substantially downregulated in patients, which was further verified by immunoprecipitation (Fig. 1B). Importantly, we did not observe any differences in the levels of SPG15 protein between Patient 1 and Patient 2, suggesting that the missense mutation carried by Patient 2 may produce a dysfunctional protein that is rapidly degraded. Every other culture showed comparable levels of SPG15, indicating that compensatory mechanisms in carrier cells may allow the less abundant *SPG15* mRNA to be translated at higher rates in order to achieve physiological levels of protein. Alternatively, the produced protein may undergo reduced turnover rates. SPG15 interacts in an ~1:1 stoichiometry with a protein called SPG11 to act as a scaffold supporting the recruitment and organization of AP-5 (a heterotetrameric complex composed of the two large subunits β5 and ζ, the medium subunit μ5 and the small subunit σ5) (12). Because it was previously demonstrated that knocking down SPG15 protein results in loss of SPG11 and destabilization of AP-5, we sought to recapitulate these observations. Expectedly, both SPG11 (Supplementary Material, Fig. S1C) and the AP-5ζ subunit (Fig. 1C) were significantly decreased in patient-derived fibroblasts.

Since it has been reported that HSP15 patients exhibit lysosomal pathology linked to loss of the AP-5 protein complex (6), we next aimed to evaluate lysosomal phenotypes in patient-derived fibroblasts. Because fibroblasts from elderly people retain hallmarks of aging on a variety of cellular parameters, including endo-lysosomal function (13,14), we excluded them from further analysis, while focusing on cultures from young individuals (i.e. Control 1, Patient 1 and Patient 2). To visualize lysosomes, we administered Lysotracker Red DND-99 (LTR) to the culture medium. LTR is a fluorescent dye that permeates the cell membrane and is selectively incorporated within acidic organelles (pH ~4.5–5.5), allowing for live or post-fixation analysis. We first imaged individual cells using high-resolution methods and observed that patient fibroblasts exhibited aberrantly enlarged acidic organelles (average of Control 1 = 0.27 μm^2^, Patient 1 = 0.44 μm^2^, Patient 2 = 0.46 μm^2^) (Fig. 1D). Here, enlargement was clearly visible in about 30% of cultured cells (Supplementary Material, Fig. S1D). We further noticed that aberrant lysosomes had a distinct perinuclear localization, in contrast to healthy lysosomes, which were typically dispersed throughout the cell body. In agreement with this observation, the average organelle distance from the cellular centroid was shorter in patients (~107 μm) than in controls (~142 μm) (Fig. 1E). Previous evidence suggests that lysosomes are highly dynamic organelles whose movement depends on their cytoplasmic location. In general, they can be classified into two types: (i) a static pool located around the microtubule-organizing center and (ii) a highly motile pool patrolling the cell’s periphery (15). Because of the characteristic perinuclear localization of lysosomes in HSP15 patient fibroblasts, we sought to determine whether these positional differences could translate into altered lysosomal dynamics. Indeed, we found that enlarged perinuclear lysosomes were more static than their healthy counterparts. In particular, they were significantly slower and prone to covering shorter distances (Fig. 1F, Supplementary Material, Videos 1–4).

Despite the evident lysosomal abnormalities in HSP15 patient cells, unstressed fibroblasts did not show any detectable changes in viability under standard culture conditions. Thus, to uncover potential mechanisms of intrinsic vulnerability, we challenged fibroblasts with chloroquine. Chloroquine blocks autophagic flux by inhibiting autophagosome–lysosome fusion and disrupting lysosome acidification. Adding 50 μm chloroquine for a period of up to 9 h induced a significant rise in the percentage of patient cells that stained positive for cleaved-caspase 3 (CC3), a known apoptotic marker (Fig. 1G). This suggested that HSP15 fibroblasts are more vulnerable under lysosomal-associated stress. Taken together, we confirmed that lysosomal aberrations are a robust hallmark of SPG15 protein depletion and that cells lacking SPG15 are more susceptible to stress.

### Lysosomal aberrations are recapitulated in cortical neurons from SPG15 knock-out mice

**Figure 2:**
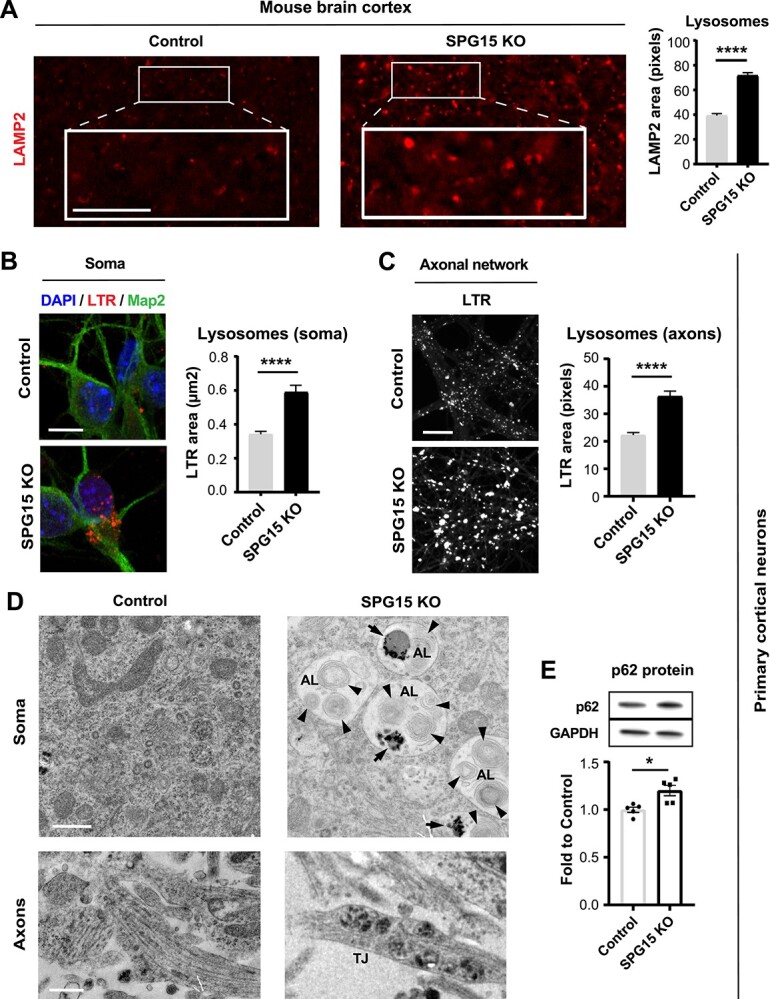
Lysosomal aberrations are an early event in disease pathogenesis. (**A**) Lysosomes are swollen in 15 month old diseased mice. The SPG15 KO brain cortex of aged mice shows significantly enlarged LAMP2-positive organelles when compared to controls. Scale bar = 100 pixels. *N* = 3 (brains, with >200 LAMP2 organelles analyzed per brain). Mann–Whitney test, error bars represent SEM. ^****^ corresponds to *P* < 0.0001. Lysosomes located (**B**) in the soma and (**C**) along the axons of cortical neurons derived from SPG15 KO mouse embryos are enlarged. Axons were separated from cell bodies by means of a microfluidic system. Confocal micrographs highlighting Lysotracker Red DND-99 (LTR)-positive organelles show a significant increase in the size of these organelles in SPG15 KO neurons. *N* = 3 (independent experiments, each evaluating up to 200 lysosomes). Scale bar = 10 μm (for soma). Scale bar = 100 pixels (for axonal network). Mann–Whitney test, error bars represent SEM. ^****^ corresponds to *P* < 0.0001. (**D**) Electron micrographs of primary cortical neurons derived from SPG15 KO and control embryos. Snapshots are taken from both cell body and axonal network. Note that the soma of SPG15 KO neurons accumulates distinct organelles consistent with ALs, harboring undigested material (black arrows) and multilamellar bodies (black arrowheads). SPG15 KO neurons also exhibit lysosomal traffic jams (TJs) along their axons. Scale bar = 500 nm. (**E**) P62 accumulates in SPG15 KO neurons. Immunoblotting shows a significant increase in diseased cells as compared to controls, suggesting a block in autophagic flux. *N* = 5. Unpaired *t*-test, error bars represent SD. ^*^ corresponds to *P* < 0.05.

HSP15 affects upper motor neurons and other cortical neuronal populations. Therefore, we were interested in recapitulating the identified phenotypes in a more relevant disease model. To this end, we aimed to analyze neurons of a well-characterized SPG15 knock-out (KO) mouse (16), by comparing SPG15 KO animals with healthy heterozygote littermates as controls, whose genotypes were confirmed by RT-qPCR in addition to genotyping (Supplementary Material, Fig. S2A). Fifteen-month-old SPG15 KO mice exhibit significantly smaller brains irrespective of their body weight (Supplementary Material, Fig. S2B). Using magnetic resonance imaging (MRI), we discovered that the reduction in brain mass is linked to a decrease in the size of both the cerebrum and the cerebellum, resulting in the formation of an unusual interspace between the two anatomical structures (Supplementary Material, Fig. S2C). The cortex of SPG15 KO mice is also significantly thinner, as determined by quantitative analysis of coronal tissue sections stained with hematoxylin/eosin (Supplementary Material, Fig. S2D). Importantly, immunostaining with anti-GFAP (Supplementary Material, Fig. S2E) and anti-IBA1 (Supplementary Material, Fig. S2F) antibodies revealed diffused astrogliosis with microglia infiltrates, demonstrating that brains of aged SPG15 KO mice undergo important pathological alterations. In agreement with our findings from patient-derived fibroblasts, SPG15 KO mice exhibited aberrantly enlarged LAMP2-positive organelles throughout the cortex (Fig. 2A) and in other brain areas (Supplementary Material, Fig. S3A). Interestingly, these structures frequently co-stained with accumulations of the intermediate filament vimentin, suggesting the presence of cytoskeletal swellings harboring enlarged lysosomes (Supplementary Material, Fig. S3B).

We next wanted to evaluate whether lysosomal pathology could be detected *in vitro* in cortical neurons from E15.5 mouse embryos. Importantly, because we compared sibling embryos, differences linked to the genetic background were strongly suppressed. To analyze lysosomal morphology in the unique polarized shape of neurons, we seeded prepped cells into microfluidic devices able to separate neuronal compartments by isolating axons from their somas. Interestingly, we found LTR-stained organelles to be significantly enlarged also in embryonic cortical neurons, indicating that lysosomal pathology occurs as a very early event in disease. In particular, we recorded a nearly 2-fold increase in the average size of lysosomes of SPG15 KO neurons compared to their healthy counterparts. This was true for both the soma (Fig. 2B) and the axonal compartment (Fig. 2C). Lysosomal swelling in SPG15 KO neurons translated into a significant increase in the intensity of LTR signal per culture (SPG15 KO average cell intensity = 10 670 a.u. versus Healthy control average cell intensity = 8466 a.u.) (Supplementary Material, Fig. S3C).

Using electron microscopy, we explored the ultrastructure of SPG15 KO and healthy neurons, which revealed that the soma of SPG15 KO neurons was constellated with large-sized organelles containing multi-lamellar bodies and undigested material, consistent with stalling ALs (Fig. 2D and Supplementary Material, Fig. S3D). Importantly, when we quantified p62 levels in these cells to assess autophagic flux, we found a mild but significant increase in p62 protein (Fig. 2E), linking SPG15 protein loss to autophagy defects. Moreover, while enlarged ALs were common in the SPG15 KO cell body, we frequently detected lysosomal buildup in SPG15 KO axonal swellings (Fig. 2D), implying a role for aberrant lysosomes in inducing axonal traffic jams.

### SPG15-KO neurons exhibit impaired axonal trafficking

**Figure 3:**
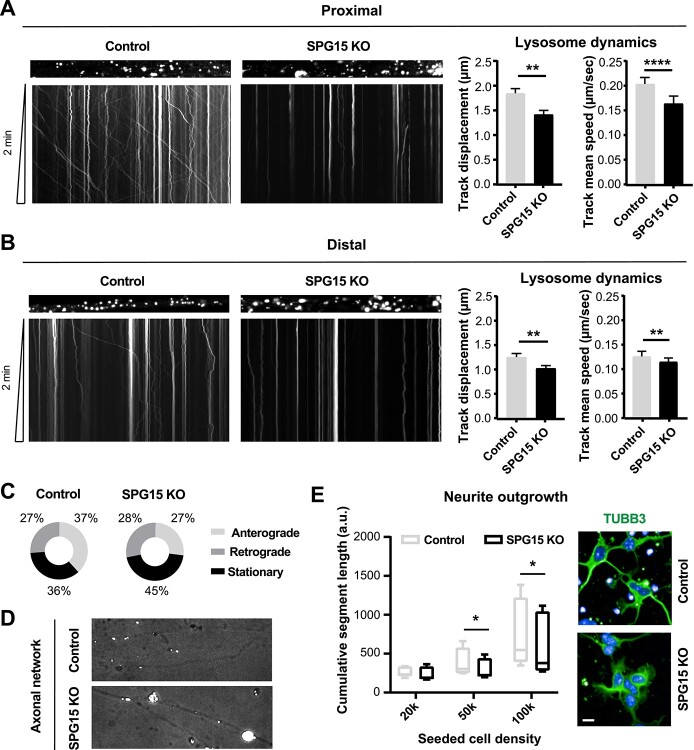
SPG15 KO neurons exhibit impaired axonal trafficking. Kymographs displaying lysosomal dynamics along (**A**) proximal and (**B**) distal axons of primary cortical neurons cultured in microfluidic chambers reveal significantly altered lysosomal shuttling in SPG15 KO cells (left), as quantified in the bar graphs (right). *N* = 3 (independent experiments, each assessing at least 10 positions). Error bars represent SEM. *t*-test. ^**^ and ^****^ correspond to *P* < 0.01 and *P* < 0.0001, respectively. (**C**) Pie charts showing the directionality of lysosomal shuttling in proximal axons. SPG15 KO neurons display an increase in the stationary fraction concomitant to a reduction in the lysosomal pool moving anterogradely. (**D**) SPG15 KO neurons show axonal swellings enriched in stalling lysosomes. Micrographs are brightfield images (highlighting the axonal network) overlayed with Lysotracker Red DND-99 fluorescent signal (labeling lysosomes). (**E**) Neurite outgrowth is hampered in SPG15 KO primary cortical neurons. The plot depicts cumulative neurite length per genotype at increasing cell densities. Example confocal micrographs show that the neurites of control neurons, stained with Tubulin Beta 3 (TUBB3), sprout more promptly. *N* = 3 (independent experiments, each evaluating at least 300 cells). Scale bar = 10 μm. *t*-test, error bars represent SEM. ^*^ corresponds to *P* < 0.05.

Given these observations, we decided to analyze the dynamics of lysosomes along the axons of primary cortical neurons. Taking advantage of the microfluidic platform, we recorded the real-time movement of LTR-labeled organelles in both the proximal and distal compartments of the axon. Lysosomes of SPG15 KO neurons were consistently less motile than controls in both the proximal (Fig. 3A) and the distal (Fig. 3B) side of the chamber (Supplementary Material, Videos 5–8). This was determined by measuring track displacement as well as track mean speed. Subsequently, we evaluated the directionality of axonal transport by quantifying the number of anterograde (moving toward the axonal terminal), retrograde (heading back to soma) and stationary lysosomes. We found that the fraction of stationary lysosomes in SPG15 KO neurons was increased by about 10%, and these lysosomes were visibly enlarged (Fig. 3C). Additionally, we observed that SPG15 KO lysosomes often stalled in traffic jams causing the formation of axonal swellings (Fig. 3D and Supplementary Material, Video 9). Importantly, anterograde transport in SPG15 KO neurons was considerably reduced, while retrograde movement was essentially unchanged (Fig. 3C).

Anterograde transport is crucial for neuronal homeostasis (17). Since neurite outgrowth requires efficient anterograde transport of proteins, lipids, organelles and vesicles (18), we monitored this process in freshly plated cortical neurons. We observed that neurite outgrowth was generally promoted by increasing cell density (Fig. 3E). This was expected, being that this process is largely driven by the neurotrophic factors released by cultured neurons themselves (19). Notably, our analysis also revealed that normalized cumulative neurite length was significantly reduced in SPG15 KO neurons, suggesting potential defects of anterograde transport in these cells (Fig. 3E). To summarize, we showed that loss of SPG15 protein is linked to lysosomal enlargement and decreased motility. In addition to lysosomal trafficking, there appears to be generalized defects in the axonal transport machinery of HSP15 neurons.

### Lysosomal aberrations are linked to lipid imbalances

**Figure 4:**
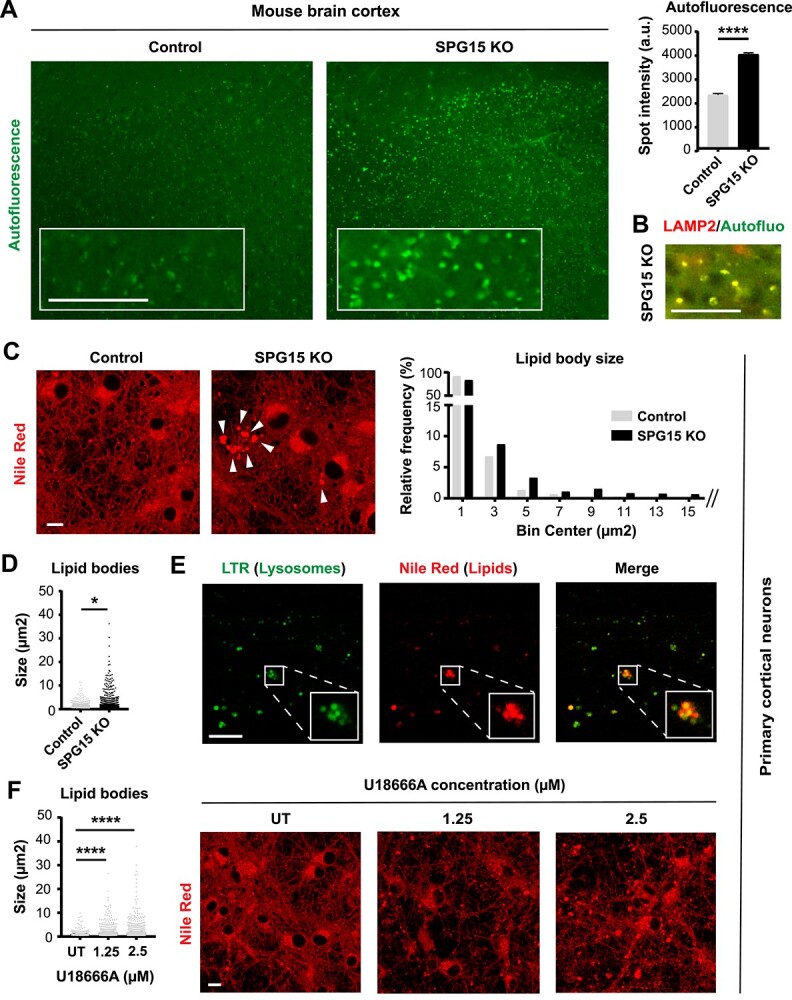
Loss of SPG15 impairs lysosomal lipid metabolism. (**A**) 15 month old SPG15 KO mice accumulate prominent autofluorescent particles throughout their cortex. Scale bar = 100 pixels. *N* = 3 (brains, with >1000 granules analyzed per brain). Mann–Whitney test, error bars represent SEM. ^****^ corresponds to *P* < 0.0001. (**B**) Autofluorescent particles co-localize with LAMP2, suggesting that these structures are lipofuscin granules. Co-localization is evident from the yellow color generated by the overlap of red (LAMP2) and green (autofluorescence) pixels. Scale bar = 100 pixels. (**C**) Loss of SPG15 protein promotes the accumulation of lipid bodies (white arrowheads) in cultures of embryonic primary cortical neurons. Lipids were stained with Nile Red. Scale bar = 10 μm. The histogram shows the frequency distribution of lipid bodies binned as per size (μm^2^). The size of each individual lipid droplet was also plotted separately to highlight a significant difference between these populations (**D**). *N* = 3 (independent experiments, each evaluating three fields). *t*-test, ^*^ corresponds to *P* < 0.05. (**E**) Lipid bodies (stained with Nile Red) co-localize with lysosomes (stained with Lysotracker Green DND-26, LTR) as evident from the yellow color generated by the overlap between red and green signals. Scale bar = 10 μm. Note that these confocal micrographs represent the axonal side of cultures grown in a microfluidic chamber. (**F**) Treatment of control neuronal cultures with two doses of U18666A (1.25 or 2.5 μm) recapitulates the lipid droplet phenotype observed in SPG15 KO neurons, suggesting that loss of SPG15 protein prevents the egress of lipids from lysosomes. *N* = 3 (independent experiments, each evaluating three fields of view). *t*-test, ^****^ corresponds to *P* < 0.0001. Scale bar = 10 μm. UT = untreated.

Previous studies on the SPG15 KO mouse model in question have highlighted marked accumulation of autofluorescent material within the cerebellum (16). Our analysis of coronal brain sections from 15-month-old SPG15 KO mice conformed to show a significant deposition of intracellular autofluorescent particles throughout the cortex. Although some autofluorescent material was also present in the control mouse cortex due to the advanced age of the animals, autofluorescent particles were significantly more intense in SPG15 KO animals (Fig. 4A). These particles were consistent with lipofuscin, the so-called ‘wear-and-tear’ yellow-brown pigment generated by aging and consisting of lipids and other biomolecules as residues of lysosomal digestion (20). Indeed, autofluorescent structures co-localized with the lysosomal marker LAMP2 (Fig. 4B), suggesting that lipids tend to aberrantly deposit within lysosomes in the SPG15 KO cortex *in vivo*.

We next moved to *in vitro* primary cortical neurons to investigate whether lipid alterations, similarly to lysosomal aberrations, could be detected as an early trait of HSP15 disease. When we stained our cultures with Nile Red, a hydrophobic fluorescent probe labeling neutral lipids including cholesterol, we found that SPG15 KO neurons exhibited evident buildup of lipid bodies of variable size (Fig. 4C and D). While small lipid bodies were also detected in control cells, the relative frequency of large-sized lipid bodies observed in SPG15 KO neurons was consistently higher, and so was the population average (average lipid droplet size SPG15 KO = 1.73 μm^2^ versus average lipid droplet size Control = 1.12 μm^2^, with larger SPG15 KO lipid bodies reaching up to 36 μm^2^ in surface area). Interestingly, the largest lipid bodies were axonal and co-localized with lysosomal staining (Fig. 4E, Pearson’s correlation coefficient = 0.75), indicating that lipids accumulate in at least a subset of lysosomes in SPG15 KO neurons.

Lysosomal cholesterol is continuously exported from lysosomes to the endoplasmic reticulum (ER) and the plasma membrane for recycling, storage and/or function (21). We speculated that the accumulation of lipids within lysosomes could be linked to cholesterol retention as a consequence of lysosomal dysfunction. To test this hypothesis, we mimicked lysosomal cholesterol trapping in control cultures by administering increasing concentrations of U18666A, a compound known to prevent cholesterol egress from lysosomes (22). Importantly, blocking lysosomal cholesterol release in healthy cells produced a phenotype that very much resembled that of SPG15 KO neurons, leading to the appearance of increasingly larger lipidic buildup (Fig. 4F). This experiment indicated that the accumulation of lipidic material in SPG15 KO lysosomes is due to the inability of SPG15 KO neurons to correctly handle lipids, thus classifying HSP15 as a lysosomal storage disease.

### SPG15 KO neurons exhibit glutamatergic dysfunction

**Figure 5:**
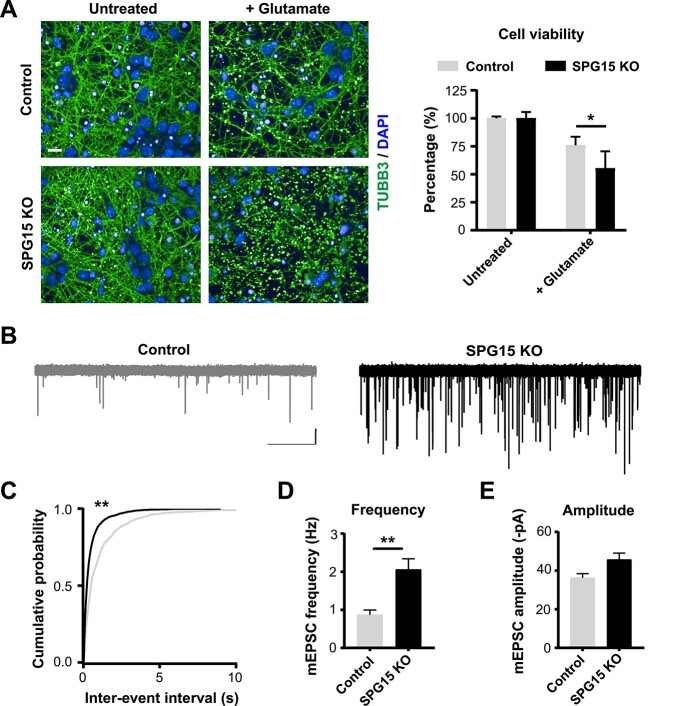
Glutamatergic dysfunction in SPG15 KO cortical neurons. (**A**) SPG15 KO neurons are more susceptible to excitotoxicity. Confocal micrographs show control and SPG15 KO primary cortical neurons subjected to 5 μm glutamate treatment. Neuronal networks of SPG15 KO cultures, stained with TUBB3, are strongly disrupted and cell viability is significantly reduced, as shown in the graph. Values are expressed as percentage to untreated. *N* = 3. *t*-test, error bars represent SEM. ^*^ corresponds to *P* < 0.05. Scale bar = 10 μm. (**B**) Sample traces from recordings of mEPSC events from control and SPG15 KO cortical neurons. Recordings were made at a holding potential of −84 mV. Scale bar = 20 pA, 5 s. Events were blocked by CNQX (10 μm) and were therefore glutamatergic (data not shown). (**C**) Cumulative probability plots of mEPSC inter-event time for control (gray) and SPG15 KO (black) cortical neurons. Kolmogorov–Smirnov test. ^***^ corresponds to *P* < 0.001. mEPSC (**D**) frequency and (**E**) amplitude for control and SPG15 KO neurons. While mEPSC frequency is significantly increased in SPG15 KO neurons, there is no change in mEPSC amplitude. *n* = 10 (cells per genotype from a minimum of three animals). *t*-test. ^**^ corresponds to *P* < 0.01.

Neurophysiological impairments remain largely unexplored in HSP, but are thought to play major pathophysiological roles in other neurodegenerative diseases. Because increased cholesterol accumulation in lysosomes is associated with basal cytoplasmic calcium elevation due to distorted store-operated calcium entry [SOCE (11)], we hypothesized that this would predispose SPG15 KO neurons to increased vulnerability to glutamate-mediated excitotoxicity, a mechanism known to depend on aberrant elevation of intracellular calcium (23). To test this possibility, we exposed our neuronal cultures to glutamate to induce calcium-mediated influx via glutamate receptor activation and evaluated their response. Of note, we observed that neuronal death triggered by glutamate exposure was moderately but significantly higher in SPG15 KO cultures (Fig. 5A). Interestingly, the observed vulnerability to glutamate was not linked to *N*-methyl-D-aspartate (NMDA) (Supplementary Material, Fig. S4A and B) nor α-amino-3-hydroxy-5-methyl-4-isoxazolepropionic acid (AMPA) receptor functional expression. The ratio of AMPA to NMDA receptor expression was also unaffected (Supplementary Material, Fig. S4A, C and D).

Because both the endosomal vesicle pathway and calcium levels are intimately associated with the supply and release of vesicular pools of synaptic transmitters (24), we considered whether synaptic transmission may also be impacted in SPG15 KO neurons. To test this, we performed whole-cell patch-clamp recordings of individual cells. Our measurements revealed an increase in the frequency, but not amplitude, of glutamatergic mini excitatory post-synaptic currents (mEPSCs) in SPG15 KO neurons (Fig. 5B–E). Our assessment of intrinsic properties indicated that this was not due to an increased maturation level of SPG15 KO neurons, since input resistance and resting membrane potential, key indicators of maturation, were comparable across genotypes. The only observed difference concerned cell capacitance, which was consistent with SPG15 KO neurons being slightly smaller than their healthy counterpart (Supplementary Material, Fig. S4E–G). The increased frequency of mEPSCs was also most likely not linked to an increase in synaptic density, as the degree of co-localization between Synapsin I (pre-synaptic marker) and PSD95 (post-synaptic marker) was essentially identical between genotypes (Pearson’s correlation coefficient SPG15 KO = 0.33 versus Pearson’s correlation coefficient Control = 0.30) (Supplementary Material, Fig. S4H). Therefore, we concluded that this phenotype was most likely due to a pre-synaptic mechanism leading to increased synaptic vesicle release.

Taken together, we showed that loss of SPG15 protein induces lysosomal storage defects that eventually culminate in synaptic dysfunction and increased vulnerability to glutamate-mediated excitotoxicity.

## Discussion

In this study, we dissected the functional consequences of SPG15 protein depletion in HSP15 disease pathogenesis, with a particular focus on the molecular mechanisms associated with lysosomal aberrations. While lysosomal pathology is an established hallmark of HSP15, the cascade of events resulting in neurodegeneration is still poorly explored. Our data highlighted a landscape of complex cellular disturbances linking lysosomal manifestations to the progressive cellular vulnerability observed in patients.

Using patient-derived fibroblasts, we recapitulated previous findings describing lysosomal enlargement in HSP15. We additionally demonstrated that enlarged lysosomes had reduced motility and abnormal perinuclear distribution. Lysosomes are the primary degradative organelles in charge of the breakdown of an assortment of macromolecules in eukaryotic cells (25). They are typically dispersed in the cytoplasm, but their subcellular localization can vary in response to perturbations, guided by the activity of kinesins and dyneins, as well as by the contacts with other organelles, such as the ER and mitochondria (26). The ability of lysosomes to flexibly shuttle throughout the cytoplasm is critical for the maintenance of cellular homeostasis, as several functions depend on correct lysosomal activity, such as autophagy. During autophagy, autophagic vesicles fuse with lysosomes giving rise to ALs, which facilitate cargo degradation and recycling (27). To increase the probability of autophagosome–lysosome fusion, this process takes place perinuclearly rather than in the cell periphery (28). Because we observed persistent perinuclear clustering in SPG15-deficient cells, this suggested that loss of SPG15 might obstruct the autophagic flux, leading to stalling of ALs. A confirmation of this came from our ultrastructural analysis of SPG15 KO primary cortical neurons, which showed evident accumulation of unresolved ALs in the neuronal soma, accompanied by increased p62 protein levels. Indeed, p62 is an autophagy receptor that is itself degraded by autophagic clearance. Hence, p62 accumulation is regarded as a general marker of hampered autophagic flux (29). Supporting our findings, silencing of SPG15 has been shown to induce accumulation of autophagy markers in a variety of cell lines (30). These results are in line with the critical role of the SPG15 protein in mediating autophagic lysosome reformation (9), which is the process leading to the regeneration of free lysosomes from ALs during autophagy. Importantly, compromised autophagic flux following SPG15 loss may increase the vulnerability of cells, such as highly specialized postmitotic neurons, to further stressors.

Similar to fibroblasts, we found that impaired lysosomal size and dynamics were obvious features of SPG15 KO embryonic cortical neurons. Particularly, we found a correlation between lysosomal enlargement and impaired lysosomal motility. We additionally observed a distinct defect in anterograde axonal transport in diseased neurons. This observation was consistent with previous reports describing axonal transport deficits in other HSPs, such as HSP type 4, the most common cause of spastic paraplegia (31). Because anterograde axonal transport defects were accompanied by delayed neurite outgrowth in SPG15 KO neurons, this indicated that axonal transport reduction induced by loss of SPG15 might not only be cargo-specific. This idea is corroborated by a recent study on HSP11, which showed that SPG11-deficient neurons were characterized by anterograde trafficking defects, downregulation of axonal genes, diminished neurite complexity and axonal instability linked to downregulation of acetylated tubulin (32). This is particularly relevant for our work since HSP11 and HSP15 are clinically indistinguishable, due to the involvement of SPG15 and SPG11 in the same molecular complex (33). We further showed that reduction in the trafficking of lysosomal organelles often resulted in axonal swellings harboring stalling lysosomes. Axonal health is a key determinant of neuronal connectivity and functionality. In fact, neurodegenerative disorders frequently manifest with axonal dysmorphia, such as swelling, that precedes axonal retraction/segmentation and, subsequently, somatic loss (34). Hence, our *in vitro* phenotype faithfully mirrors the length-dependent dying back axonopathy observed in HSP patients (35), where lower limb paralysis would be linked to disruptions in the cortico-spinal tract, while declined cognitive abilities could be attributed to progressive atrophy of the frontal cortex (36). Of note, although lysosomal pathology was prominent in the cortex of symptomatic 15-month-old SPG15 KO mice, these phenotypes were already evident in embryonic cortical neurons. Hence, we concluded that lysosomal alterations occur as an early event in disease pathogenesis, largely before clinical disease onset.

Another early trait of SPG15 KO cortical neurons was that lysosomal organelles were often enriched in droplets of neutral lipids, such as cholesterol. This was in agreement with previous findings from studies investigating HSP11, where other lipid species, including gangliosides, were additionally identified as an integral part of these deposits (37). Such lysosomal lipid bodies possibly represent the precursors of the lipofuscin granules, we found to be embedded in the cortex of aged animals. Accumulation of lipids in SPG15 KO lysosomes is reasonably due to the defects in lysosomal recycling described above, as autophagy is known to regulate lipid metabolism (38). However, it cannot be excluded that SPG15 may be involved in lipid transport, i.e. playing a role in transferring lipids between organelles and other subcellular compartments. Further experiments will elucidate whether SPG15 and/or its binding partners may be involved in this process. Lipids are amphiphilic molecules that make up cell membranes and define different subcellular domains based on their specific pattern and composition (39). Particularly, cholesterol is a critical component of the plasma membrane, and its metabolites are employed as important signal transducers (40). Therefore, preserving lipid distribution and trafficking is fundamental for cellular homeostasis, as highlighted by the role of abnormal lipid metabolism in the pathogenesis of numerous neurodegenerative disorders, including Alzheimer’s disease (41), Parkinson’s disease (42), motor neuron disease (43) and other subtypes of HSP, e.g. SPG5 (44), SPG26 (45), SPG35 (46) and SPG46 (47).

Previous clues have linked reduced cholesterol egress from lysosomes to increased intracellular calcium levels (11,48), suggesting that SPG15 KO cortical neurons may be vulnerable to glutamate-mediated excitotoxicity, of which elevated calcium influx is the toxic pathological substrate (49). Indeed, we determined that SPG15 KO neurons exhibit an increased susceptibility to glutamate excitotoxicity, an established pathophysiological mechanism for multiple neurodegenerative diseases (50). However, our data showed that the functional expression of AMPA and NMDA receptors did not differ between control and SPG15 KO neurons, suggesting that the increased vulnerability of SPG15 KO neurons to glutamate-mediated excitotoxicity may be caused by reduced capacity to buffer glutamate receptor-mediated calcium influx rather than increased AMPA/NMDA functional receptor expression. Given this vulnerability and considering that the late-endosomal pathway (that is involved in vesicle trafficking) is disrupted in HSP15, we speculated that loss of SPG15 protein may additionally impact upon synaptic activity. Interestingly, we discovered a significant increase in the frequency of mEPSC events in SPG15 KO neurons. This finding was rather unexpected since defective anterograde transport in SPG15 KO neurons would predict the presence of a reduced pool of synaptic vesicles. This piece of evidence most parsimoniously suggests that the rise in mEPSCs can only be explained by an increase in calcium-driving synaptic exocytosis. As noted above, SPG11/AP-5 defects have been shown to impair calcium homeostasis. Interestingly, neuronal lysosomes and lysosome-related organelles have been reported to act as stores of readily releasable calcium (51), and evidence suggests that calcium released from the lysosomal compartment following a variety of insults can contribute to synaptic vesicle release (52). This is because calcium released from acidic calcium stores can stimulate calcium influx across the plasma membrane via the unique mechanism of acidic store-operated calcium entry (aSOCE), which is conceptually similar to but mechanistically distinct from the classical SOCE driven by the ER (53). In line with these considerations, our results suggested that dysregulated calcium homeostasis may be the cause underlying the observed disruption in pre-synaptic vesicular dynamics, contributing to increased vesicle release probability. However, further experiments will be required to dissect calcium dyshomeostasis in this model.

Perturbations of synaptic function in the context of HSP15 have not been reported to date, and changes in synaptic properties in other HSPs have started to be characterized only recently. For instance, disrupted KIF1A signaling has been associated with increased synaptic vesicle accumulation (54), while Spastin depletion has been connected to synaptic loss (55). Overall, our data allow us to propose a model of degeneration in HSP15, where the increased pre-synaptic release of glutamate exacerbates an intrinsic vulnerability of cortical neurons to glutamate excitotoxicity, which, in turn, is linked to lysosomal dysfunction. Our results suggest that targeting lysosomal aberrations and/or glutamatergic function in HSP15 patients may represent a fascinating therapeutic avenue.

## Materials and Methods

### Ethics approval

All procedures involving human-derived samples were performed in accordance with the ethical standards of the national research committee as well as with the 1964 Helsinki declaration and its later amendments. Informed consent was obtained from all subjects before sample collection by the Queen Square Ethics Committee. Animal experiments were conducted according to the Animals Scientific Procedures Act (ASPA) of 1986. Both animal research and experiments using human-derived samples were further approved by the University of Sheffield’s Ethical Review Committee.

### Cell culture and treatments

#### Fibroblasts

Human dermal fibroblasts were obtained from skin biopsies performed at University College London and cells were kindly provided by Professor Henry Houlden (UCL, London, UK). Fibroblasts were cultured in DMEM high glucose (BE12-741F, Lonza, Basel, Switzerland) supplemented with 10% FBS (10500-064, Gibco, Waltham, Massachusetts, United States), 1% Pen/Strep (15140122, Thermo Fisher Scientific, Waltham, Massachusetts, United States) and 50 mg/ml uridine (A15227, Alfa Aesar, Ward Hill, Massachusetts). For passaging, cells were briefly incubated with HBSS medium (H9394, Sigma, Burlington, Massachusetts, United States) supplemented with 50 mm EDTA (ED2SS, Sigma, Burlington, Massachusetts, United States) and subsequently dissociated using trypsin (BE02-007E, Lonza, Basel, Switzerland). Cells were maintained in T75 flasks and typically split twice a week for expansion. No experiment was performed beyond P30. Treatment with chloroquine (C6628, SigmaBurlington, Massachusetts, United States) was performed at a concentration of 50 μm. Fibroblasts included two SPG15 patient lines (Patient 1: female, 15 years old at biopsy, 4312C>T and 837T>G mutations; Patient 2: male, 19 years old at biopsy, 7041C>A and 5822T>C mutations), two heterozygotes (Carrier 1: female, 48 years old at biopsy; Carrier 2: male, 59 years old at biopsy) and two healthy controls (Control 1: female, 20 years old at biopsy; Control 2: male, 59 years old at biopsy).

#### Primary cortical neurons

Cultures of primary cortical neurons were isolated from E15.5 embryos of ZFYVE26^+/−^ (heterozygote) mice (16). Briefly, brains were removed and transferred to HBSS^−/−^ medium (14170-088, GibcoWaltham, Massachusetts, United States). Under a dissection microscope, the hemispheres were separated and depleted from the meninges and the midbrain. Next, the isolated cortical tissue was incubated with trypsin (15090-046, Gibco, Waltham, Massachusetts, United States) for dissociation and triturated into a single-cell suspension mechanically by pipetting it in trituration solution. This consisted of HBSS+/+ (14025–092, GibcoWaltham, Massachusetts, United States) supplemented with 1% Albumax (11020-013, Gibco, Waltham, Massachusetts, United States), 25 mg Trypsin inhibitor (T9003, Sigma), 1% DNase 10 mg/ml (D5025, Sigma, Burlington, Massachusetts, United States). Cortical neurons were resuspended in neuronal medium, made up of Neurobasal medium (11570556, ThermoFisher Scientific) supplemented with B27 (17504-044, Gibco), 1% Pen/Strep (15140122, ThermoFisher Scientific) and 1% glutamine (BE17-605E, Lonza). Cells were seeded onto tissue culture plates previously coated with poly-d-lysine (P6407, Sigma). Unless otherwise stated, for 96-well plates, 60k cells/well were seeded. For six-well plates, 1.5 million cells/well were seeded. Neurons were kept in culture at 37°C in the presence of 5% CO_2_ for 10–12 days prior to analysis. On alternative days, half of the medium was replaced with fresh medium. Treatment with 5 μm l-glutamic acid (Glutamate, G1251, Sigma) was performed for 24 h.

### Microfluidics

Axonal trafficking experiments were performed using live primary cortical neurons plated into silicone round microfluidic devices endowed with a 900 μm microgroove barrier and an approximate diameter of 21 mm (RD900, Xona, Durham, North Carolina, United States) mounted onto 35 mm glass-bottom dishes (FD35-100, Fluorodish) following poly-d-lysine coating. A total of 400k cells were plated on the soma side of the chamber (200k/well). While the soma side was fed every second day with plain neuronal medium, the axonal side was supplemented with 10 ng/ml GDNF (450-10-10, Peprotech, Rocky Hill, New Jersey, United States) and 10 ng/ml BDNF (PHC7074, Peprotech) to establish a chemoattractive gradient and isolate axons due to the microgrooves being too small to be penetrated by the larger neuronal bodies. Neurotrophic factors were removed on the last medium change prior to live imaging to prevent their interference with the axonal trafficking readout.

Lysosomes of live cells were stained with 1 μm Lysotracker Red DND-99 (LTR, L7528, ThermoFisher Scientific) by diluting the dye in culture medium and letting it permeate the cells for 45 min before live imaging. Both the soma and the axonal side were fed with a dye-containing medium. Live-cell imaging was performed at the proximal and distal side of the microgrooves, but not in the very center, as the stain does not easily penetrate. LTR signal is also largely maintained after fixation with 4% paraformaldehyde (PFA) in phosphate buffered saline (PBS). Hence, in order to combine LTR staining with immunocytochemistry, we simply fixed the cells post LTR addition and proceeded with a standard ICC protocol as described in the paragraph ‘Staining of cultured cells’.

### Mouse genotyping

Genotyping was performed from either ear clips of young mice (for breeding) or from tail tips of embryos (for cortical prep). Briefly, genomic DNA was extracted by incubating the tissue in QuickExtract™ DNA Extraction Solution (QE09050, Lucigen, Middleton, Wisconsin, United States) at 65°C for 15 min, followed by 2 min at 98°C. One microliter of extracted DNA was used for PCR amplification with 5× FIREPol® Master Mix Ready to Load (04-12-00115, Solis BioDyne, Tartu, Estonia). The following primers were combined in two parallel reactions: FW = *5′-*CTTGTGTATTTTGCATAGGTGC-*3′*, REV = *5′-*TGACACTGAATGTTAAGA-*3′* and Del = *5′-*TTCTGGAAGCGTCTGTAAAG*-3′* were combined in one PCR mix; the primers FW2 = *5′-*GGTACTCATGTTCAACCTGA*-3′* and REV2 = *5′-*ATGGCCTGGAGAGTTGAG*-3′* were used for the second mix. The primer set FW/REV/Del amplified a 133 bp band from the WT allele and a 250 bp band in SPG15 KO or heterozygous mice. The primer pair FW2/REV2 amplified a 180 bp band in WT and heterozygous mice, but not in SPG15 KOs. After an initial denaturing step at 95°C for 3 min, 30 PCR cycles were performed using the following scheme: 15 s at 95°C for denaturation, 30 s at 56°C for annealing and 45 s at 72°C for amplification. PCR products were run on a 2% agarose gel.

### Magnetic resonance imaging

At postnatal day 450 (15 months of age), cohorts of SPG15 KO and heterozygous mice were perfused with PBS and fixed with 4% PFA (P6148, Sigma). Brains were extracted leaving out the olfactory bulb and stored in fixative solution overnight. Next, fixed brains were washed three times in PBS and weighted using an OHAUS Pioneer analytical balance prior to MRI. All scanning was performed on a 9.4 T MRI (Bruker Avance III spectrometer, 44 mm diameter vertical bore, 1500 mT/m gradient strength, Bruker). Each brain was placed into a 3D-printed plastic cradle such that the long axis of the brain was perpendicular to the main magnetic field. This was held in place with another 3D-printed plastic cap. The brain and cradle were inserted into a glass vial that was covered with plastic film. The brain was positioned so as to be located at the center of a 25 mm 1H volume rf coil (Bruker). After obtaining scout scans, high-resolution spin echo images were acquired in the coronal plane according to the following parameters: rapid acquisition with refocused echoes (RARE) 50 × 50 μm in plane resolution; 500 μm slice thickness; time to echo (TE) 20 ms; rapid acquisition with refocused echoes (RARE) factor 4; Effective TE 40 ms; repetition time (TR) 2500 ms; NA 32; fields of view (FOVs) were chosen to achieve in plane resolution—typically: 15–17 × 12 mm; matrix size 300–340 × 240, 15–19 contiguous slices; scan time ~80  min. The imaging plane was orientated such that the slices were parallel with the corpus callosum, with one of the slices aligned to this structure. After reconstruction using Paravision 5.1 (Bruker), all images were converted to tiff format (Custom Matlab script, Mathwork, Natick) for analysis in ImageJ.

### Immunohistochemistry of mouse tissue

Following MRI, brains were transferred to 15% sucrose (S24060, Melford, Ipswich, United Kingdom) in PBS until they sank completely and subsequently to 30% sucrose. Brain tissue was frozen using isopentane (C5H12, Acros Organics, Geel, Belgium) immersed in liquid nitrogen and transferred to a −80°C freezer prior to sectioning. Sectioning was carried out using a Leica CM3050S cryostat to produce 30-μm-thick coronal slices collected in free-floating in PBS supplemented with 1.5 mm sodium azide. Immunofluorescence staining of brain sections was performed as indicated below for cultured cells. However, primary antibodies were applied for 2 h at room temperature (RT). These included rabbit anti-GFAP (z0334, Dako) 1:1000, mouse anti-LAMP2 (sc-18 822, Santa Cruz) 1:400 and chicken anti-Vimentin (AB5733, EMD Millipore) 1:1000. Sections were mounted onto charged glass slides (MBB-0302-55A, Starfrost, Lowestoft, United Kingdom) in Fluoromount Aqueous Mounting Medium (F4680, Sigma). For Hematoxylin/Eosin, floating sections were transferred to a charged glass slide and allowed to dry out. Slides were rehydrated in 95% alcohol for 5 min, followed by 5 min in 70% alcohol. Subsequently, they were dipped in tap water and Hematoxylin (RBA-4205-00A, Cellpath, newton, United Kingdom) solution for 2 min. After another wash in tap water, sections were dipped in acid alcohol solution, washed in tap water and transferred to Scott’s water for up to 60 s. After a washing step in tap water, incubation in Eosin solution (3801590E, Leica, Wetzlar, Germany) was done for 5 min, followed by a tap water wash, dehydration in alcohols (70%, 90%, 100%) and storage in xylene (X/0200/17, Fisher) until mounted using DPX mounting medium (SEA-1300-00A, Cellpath, Newton, United Kingdom). DAB staining was performed using ABC DAB (SK-4100, Vector Laboratories, Burlingame, California, United States) and Elite ABC Kit Vectastain (PK-6101, Vector Laboratories, Littleton, Colorado, United States) according to the manufacturer’s instructions. The primary antibody goat anti-IBA-1 (NB100-1028, Novus Biologicals) 1:250 was applied for 2 h at RT. Eventually, nuclei were counterstained with Hematoxylin, and slides were mounted as described above.

### Staining of cultured cells

Lysosomes were stained with 1 μm Lysotracker Red DND-99 (LTR, L7528, ThermoFisher Scientific) or Lysotracker Green DND-26 (L7526, ThermoFisher Scientific) by diluting the dye in culture medium and letting it permeate the cells for 45 min prior to fixation/live imaging. Note that for axonal trafficking experiments, both the soma and the axonal side were fed with dye-containing medium. Lipid bodies were stained with Nile Red (22 190, AAT Bioquest, Sunnyvale, California, United States), which was added to the culture medium for 15 min prior to fixation at a concentration of 1 μm. Cells were fixed with 4% PFA (P6148, Sigma, Burlington, Massachusetts, United States) in PBS for 25 min. Permeabilization and blocking were performed in a single step by treating cells with PBS supplemented with 0.1% Triton (A16046, Alfa Aesar, Ward Hill, Massachusetts), 10% FBS (10500–064, Gibco) and 1% BSA (5217, Tocris, Bristol, United Kingdom) for 45 min. Cells were incubated with primary antibodies overnight at 4°C. Secondary antibodies were applied for 1 h at RT. All antibodies were diluted in 0.1% BSA in PBS to reduce non-specific binding. Finally, cells were incubated for 5 min with Hoechst in PBS. Washes between each step were done using 0.1% BSA in PBS. Cells were left in PBS for imaging. Primary antibodies included rabbit anti-cleaved caspase 3 (9661S, Cell Signaling, Danvers, Massachusetts, United States) 1:400, mouse anti-MAP2 (M4403, Sigma) 1:500, mouse anti-Tuj1 (801 201, Biolegend, San Diego, California, United States) 1:1000, mouse anti-PSD95 (MAB1596, Millipore, Burlington, Massachusetts, United States) 1:500 and rabbit anti-Synapsin 1 (S193, Sigma) 1:1000. Secondary antibodies included AlexaFluor 488, 568 and 647 (ThermoFisher Scientific), all 1:1000.

### Light microscopy and image analysis

#### Fixed cells/tissue

Fixed cells were imaged using a confocal Zeiss LSM 880 with Airyscan detector (63× objective, oil immersion NA 1.4) for improved lysosome resolution, a Leica SP5 confocal microscope (63× objective, oil immersion NA 1.4) for standard cell imaging or an Opera Phoenix (40× objective, water immersion NA 1.1) for high content studies. For immunofluorescence, mouse brain tissue was imaged using a Nikon Eclipse fluorescent microscope (20× objective, air NA 0.7). Alternatively, brain sections stained with Hematoxylin/Eosin and DAB were imaged using a Nanozoomer-XR scanner (Hamamatsu, Hamamatsu, Japan). Lysosome size was calculated using Fiji. Following maximum projection and conversion to binary image for segmentation, lysosome area was obtained using the command ‘analyze particles’ and the following parameters: size = 0.1–infinity, circularity = 0.3–1.0. Lipid droplet size was determined in a similar fashion. The subcellular localization of lysosomes was determined using CellProfiler. Briefly, the relative distance between each lysosome and the cell centroid was calculated after segmenting lysosomes (child objects) and relating them to their respective cell (parent object). Measurements were obtained as child–parent distances. Only cells of a similar size and shape were analyzed for consistency. The percentage of CC3-positive cells was assessed using the Columbus™ Image Analysis System provided by PerkinElmer. CC3-positive cells were defined as objects exhibiting cytoplasmic signal intensity superior to baseline noise. The measurement was expressed as percentage over total. Neurite outgrowth was measured in an automated manner using the Columbus™ Image Analysis System. This was done by segmenting neurites using the cytoskeletal marker Tuj1 and selecting the ‘Find neurites’ command. LTR intensity in neurons was quantified using Columbus™ by segmenting the cell soma and assessing absolute LTR intensity within the cell body, nucleus excluded. The percentage of cells exhibiting high LTR was determined based on an arbitrary threshold that was calculated as the difference between the means of the two groups under comparison. Neuronal survival after glutamate treatment was measured by identifying nuclei with Columbus™ and normalizing values to control conditions. Co-localization was assessed using the Coloc2 function of Fiji and expressing correlation as Pearson’s *R* value. For mouse brain volumetric studies after MRI, cerebrum and cerebellum size were determined by outlining their borders and measuring the area comprised within the selection. The cortical thickness of Hematoxylin/Eosin section was determined using the ruler function of the NanoZoomer Digital Pathology software. DAB-stained sections were analyzed using QuPath Quantitative Pathology & Bioimage Analysis software (56). Briefly, the Hematoxylin and DAB signals were separated using the QuPath color transforms function. Cortical cells were identified by running the ‘Cell Detection’ command after manually drawing a region of interest around the cortex. Default settings were maintained. Subsequently, DAB-positive cells were detected within selected cortical cells using the ‘Positive Cell Detection’ command.

#### Live-cell imaging

Live imaging of lysosomal dynamics in fibroblasts was performed using a confocal Zeiss LSM 880 microscope with Airyscan detector (63× objective, oil immersion NA 1.4). Here, lysosomal dynamics were acquired for 3 min and subsequently analyzed using the ‘TrackMate’ plugin of Fiji: lysosomes were segmented using the DoG detector (0.5 μm size for control, 1 μm size for patients) and traced via Simple LAP tracker. Analysis was run on track results. Live imaging of lysosome dynamics in cortical neurons was carried out using a Perkin Elmer Spinning Disk confocal microscope (60× objective, oil immersion, NA 1.4). Imaging was performed at the extremities of the microgrooves because the LTR signal became too diluted in the very center of the device due to reduced penetration of the dye. Lysosomal dynamics along proximal and distal axonal segments were recorded for 2 min, and traces were analyzed using the Multikymogram function of Fiji to produce kymographs. Obtained kymographs were run through the Kymobutler software (57) using default settings except for the following parameters: pixel size = 0.23 μm and frame rate = 0.17 s. Plots and statistical analysis were generated using Graphpad Prism.

### Electron microscopy

Neurons were grown on u-Dish 35 mm high grid-500 glass-bottom Ibidi culture plates (IB 81168, Thistle Scientific) at a density of 700k cells/plate. Cells were fixed using 2.5% glutaraldehyde/0.1 m sodium cacodylate buffer for 2 h. Next, the fixative was replaced with buffer solution, and cells were further fixed using 1% aqueous osmium tetroxide (AGR1024, Agar Scientific, Essex, United Kingdom) for 2 h. Samples were dehydrated in a series of ethanol solutions (50, 75, 95, 100%) for a minimum of 5 min per solution. Dried ethanol was eventually replaced with intermediate solvent epoxy propane (EPP, R1080, Agar Scientific, Essex, United Kingdom) for no longer than 5 min. Pure EPP was substituted with a 50:50 mixture of EPP and Araldite resin—a mix of CY212 and DDSA plus BDMA (AGR1040, AGR1051, AGR1060, all Agar Scientific) for 30 min and subsequently exchanged with fresh Araldite resin (Agar Scientific) for 1 h. EM blocks were cast by inverting BEEM capsule mold (G360–1, Agar Scientific) over the central region of the plate grid, followed by curing in a 60°C oven for 48 h. After removal, the block was removed by heat-shocking in boiling water followed by immersing in liquid nitrogen. The block was then sectioned using a Reichert-Jung ultramicrotome and diamond knife (Diatome) at ~85 nm. Sections were collected onto 300 mesh nickel grids (G2300n, Agar Scientific) and stained using 1% uranyl acetate (U001/S/2/25, TAAB Laboratories, Aldermaston, United Kingdom) and Reynold’s lead citrate. Samples were viewed using a Tecnai spirit T-12 transmission electron microscope at an operating voltage of 80 kV and recorded using a Gatan Orius 1000B CCD digital camera and Gatan digital micrograph software.

### Patch-clamp electrophysiology

On the evening prior to patch-clamping, the neuronal culture medium was replaced with SCG/MEM medium. This consisted of 90% SCG medium and 10% MEM medium (11095080, Gibco). SCG medium was prepared in house based on the following recipe: 114 mm NaCl, 0.219% NaHCO_3_, 5292 KCl, 1 mm MgCl_2_, 2 mm CaCl_2_,10 mm HEPES, 1 mm Glycine, 30 mm Glucose, 0.5 mm NaPyr, 0.1% Phenol Red, pH = 7.4 and osmolarity = 340 mOsm. Whole-cell voltage-clamp recordings were performed as previously described (58) using electrodes filled with (in mM): 155 K-gluconate, 2 MgCl_2_, 10 Na-HEPES, 10 Na-PiCreatine, 2 Mg_2_-ATP and 0.3 Na_3_-GTP, pH 7.3, 300 mOsm. Cells were bathed in an extracellular recording solution comprising (in mm): 152 NaCl, 2.8 KCl, 10 HEPES, 1.5 MgCl_2_; 2 CaCl_2_, 10 glucose, pH 7.3, 320–330 mOsm that was also supplemented with TTX (300 nm) and picrotoxin (50 μm). The extracellular solution was supplemented with glycine (50 μm) and had MgCl_2_ omitted when recording NMDA-mediated currents. Current recordings were typically low-pass filtered online at 2 kHz, digitized at 10 kHz and recorded to the computer using the WinEDR V2 7.6 Electrophysiology Data Recorder (J. Dempster, Department of Physiology and Pharmacology, University of Strathclyde, UK). Series resistance compensation was applied up to 75%. Recordings were omitted from analysis if the series resistance changed by more than 20% during the experiment, or if they exceeded 20 MΩ. mEPSC recordings were analyzed offline using the WinEDR software stated above. A dead time window of 10 ms was set and individual mEPSCs were detected using an algorithm that selected for mEPSCs above a −6 pA amplitude threshold and greater than 1 ms in duration. mEPSCs that had a monotonic rising phase with a 10–90 rise time of lower than 6 ms and a Τ-decay with a decay time constant of lower than 25 ms were selected for analysis. Recordings were then visually inspected for validity. mEPSC data were obtained from at least 2-min recordings, and neurons that displayed mEPSC frequencies under 0.1 Hz were omitted from the analysis. Statistical analysis was performed using GraphPad Prism.

### Immunoprecipitation

Endogenous SPG15 was immunoprecipitated using the ab206996 Immunoprecipitation kit (Abcam, Cambridge, United Kingdom). Briefly, cells were lysed for 30 min in ice-cold non-denaturing lysis buffer supplemented with the protease inhibitors provided. Lysates were cleared at 15000*g* for 20 min at 4°C. Protein concentrations were determined by BCA (1859078, ThermoFisher Scientific); 250 μg of sample were incubated with rabbit anti-SPG15 (8532, Cell Signaling) at a 1:50 dilution. Incubation was carried out overnight at 4°C on a rotary mixer. On the following day, the solution was mixed with 25 μl of Protein A/G Sepharose® beads. Beads coated with the antigen–antibody complex were collected by low-speed centrifugation (2000*g* for 2 min). The complex was eluted by brief incubation with 40 μl of 0.2 m Glycine pH 2.5, followed by centrifugation and addition of 4 μl 1 m Tris base pH 10.4 for neutralization. Eluates were further processed for immunoblotting.

### Immunoblotting

Protein lysates were generated by harvesting cells in ice-cold RIPA buffer [50 mm Tris–HCl pH 6.8, 150 mm NaCl, 1 mm EDTA, 1 mm EGTA, 0.1% (w/v) SDS, 0.5% (w/v) sodium deoxycholate, 1% (w/v) Triton X-100 + protease inhibitor cocktail]. Lysates were cleared through a centrifugation step at 15000*g* for 20 min, 4°C. Protein concentrations were determined by BCA (1 859 078, ThermoFisher Scientific). 10–20 μg of each protein lysate were mixed with 5× Laemmli buffer and loaded onto 4–20% Mini-PROTEAN® TGX™ Precast Protein Gels (4 568 094, Biorad, Hercules, California, United States) after denaturation at 95°C for 5 min. Gels were run at 110 V for 70 min. Blotting was performed on 0.2 μm nitrocellulose membranes (1704158, BioRad) using a Trans-Blot Turbo Transfer System (1704150, BioRad), 1 Mini-TGX protocol. Next, membranes were blocked for 1 h in 5% milk in TBS-T and incubated with primary antibodies overnight at 4°C. After multiple washes, membranes were incubated with HRP-coupled secondary antibodies and developed with enhanced chemiluminescent ECL solution (RPN2232, GE Healthcare, Chicago, Illinois, United States). Signal was detected with a LI-COR image analyzer. Primary antibodies included rabbit anti-SPG15 (8532, Cell Signaling) 1:500, rabbit anti-SPG11 (16555-1-AP, ProteinTech, Rosemont, Illinois, United States), rabbit anti-AP-5ζ (HPA035693, Sigma) 1:500, mouse anti-GAPDH (CB-1001, Merck Millipore) 1:3000 and mouse anti-Tubulin (T9026, Sigma) 1:2000. Secondary antibodies included anti-mouse HRP (31430, Invitrogen, Waltham, Massachusetts, United States) 1:3000 and anti-rabbit HRP (12-348, Merck Millipore) 1:3000. Images were analyzed with Fiji and plotted using GraphPad Prism.

### RNA extraction and RT-qPCR

RNA was extracted using the RNeasy Mini Kit (74106, Qiagen, Hilden, Germany) following the manufacturer’s instructions with the following highlights: lysis of cultured cells was performed by directly dispensing 350 μL RLT buffer onto the monolayer and mechanically disrupting cells by pipetting. For mouse brains, 20 μg of tissue was cut and dipped into 450 μl RLT buffer, followed by homogenization using a Precellys Evolution tissue homogenizer, program details: 5500 rpm, 2 cycles of 30 s each with a 20 s pause in between. The program was repeated twice. During RNA extraction, an on-column DNA digestion step using the RNase-Free DNase Set (79254, Qiagen) was included. Next, isolated RNA was diluted to a concentration of 10 ng/μl and reverse-transcribed plus amplified in a single reaction using the QuantiFast® SYBR® Green RT-PCR kit (204 054, Qiagen). RT-qPCR was performed using a Biorad CFX96 C1000 Touch machine. Cycling conditions included a reverse transcription step for 10 min at 50°C, followed by an initial PCR activation step of 5 min at 95°C. This was followed by a two-step cycling protocol, including a denaturation step at 95°C for 10 s and a combined annealing/extension step at 60°C for 30 s. A total of 40 cycles were run. Fold change in gene expression was calculated using the double Δ Ct method. The list of RT-qPCR primers used in this study is as follows: hSPG15 FW = 5′-GCTCTTTGTGACAGCTACATCA-3′, hSPG15 REV = 5′-AGTTGGTAGTACTCGGCTTCC-3′, hGAPDH FW = 5′-CTGGTAAAGTGGATATTGTTGCCAT-3′, hGAPDH REV = 5′-TGGAATCATATTGGAACATGTAAACC-3′, mSPG15 FW = 5′-CGTTGCTTTCCAGATACGCA-3′, mSPG15 REV = 5′-CGGAGAACTCAGCTGACGAT-3′, mβ-actin FW = 5′-ATCTGGCACCACACCTTC-3′, mβ-actin REV = 5′-AGCCAGGTCCAGACGCA-3′.

## Supplementary Material

Supplementary_Figure_1_ddac063

Supplementary_Figure_2_ddac063

Supplementary_Figure_3_ddac063

Supplementary_Figure_4_ddac063

Supplementary_Video_1_ddac063

Supplementary_Video_2_ddac063

Supplementary_Video_3_ddac063

Supplementary_Video_4_ddac063

Supplementary_Video_5_ddac063

Supplementary_Video_6_ddac063

Supplementary_Video_7_ddac063

Supplementary_Video_8_ddac063

Supplementary_Video_9_ddac063

HMG-2022-CE-00021_Marrone_et_al_2021_Formatted_Supplementary_files_ddac063
