# Supplementary material for: SPG15 protein deficits are at the crossroads between lysosomal abnormalities, altered lipid metabolism and synaptic dysfunction
Source: Hum Mol Genet. 2022 Mar 21;31(16):2693–710. doi: 10.1093/hmg/ddac063 (PMC9402239; doi:10.1093/hmg/ddac063)
Supplement: HMG-2022-CE-00021_Marrone_et_al_2021_Formatted_Supplementary_files_ddac063 [file hmg-2022-ce-00021_marrone_et_al_2021_formatted_supplementary_files_ddac063.zip › HMG-2022-CE-00021_Marrone_et_al_2021_Formatted_Supplementary_files_ddac063.docx]

**Supplementary Figures**

**
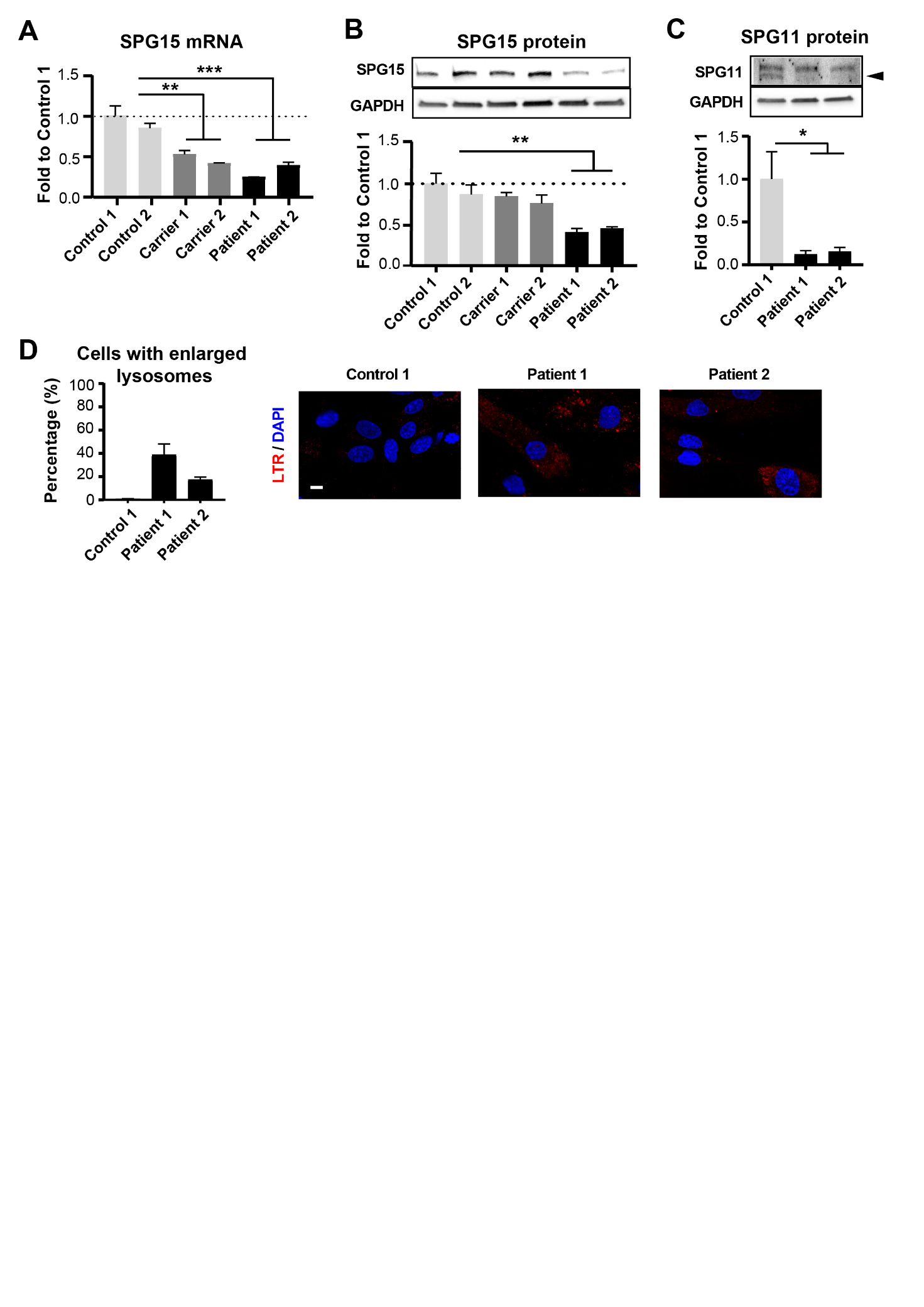
Supplementary Figure 1. Characterization of patient fibroblasts. Related to Figure 1.** A) *SPG15* mRNA levels are reduced in the presence of nonsense mutations. RT-qPCR analysis shows a reduction of *SPG15* transcript abundance in carriers and patients. Note that Patient 2 carries a combination of missense/nonsense. N = 3, Unpaired t-test. Error bars represent SD. ** and *** correspond to p<0.01 and p<0.001, respectively. B) Immunoblotting shows a significant deficiency of SPG15 protein in patients compared to carriers and controls. N = 3 Unpaired t-test, error bars represent SD. ** corresponds to p<0.01. C) Immunoblotting shows a significant deficiency of SPG11 protein (black arrowhead) in patients compared to controls. N = 3 Unpaired t-test, error bars represent SD. * corresponds to p<0.05. D) The phenotype of lysosomal enlargement is not homogeneous within the culture. Confocal micrographs and respective quantifications reveal that only a fraction of patient fibroblasts contains aberrantly enlarged lysosomes. Scale bar = 10 µm.


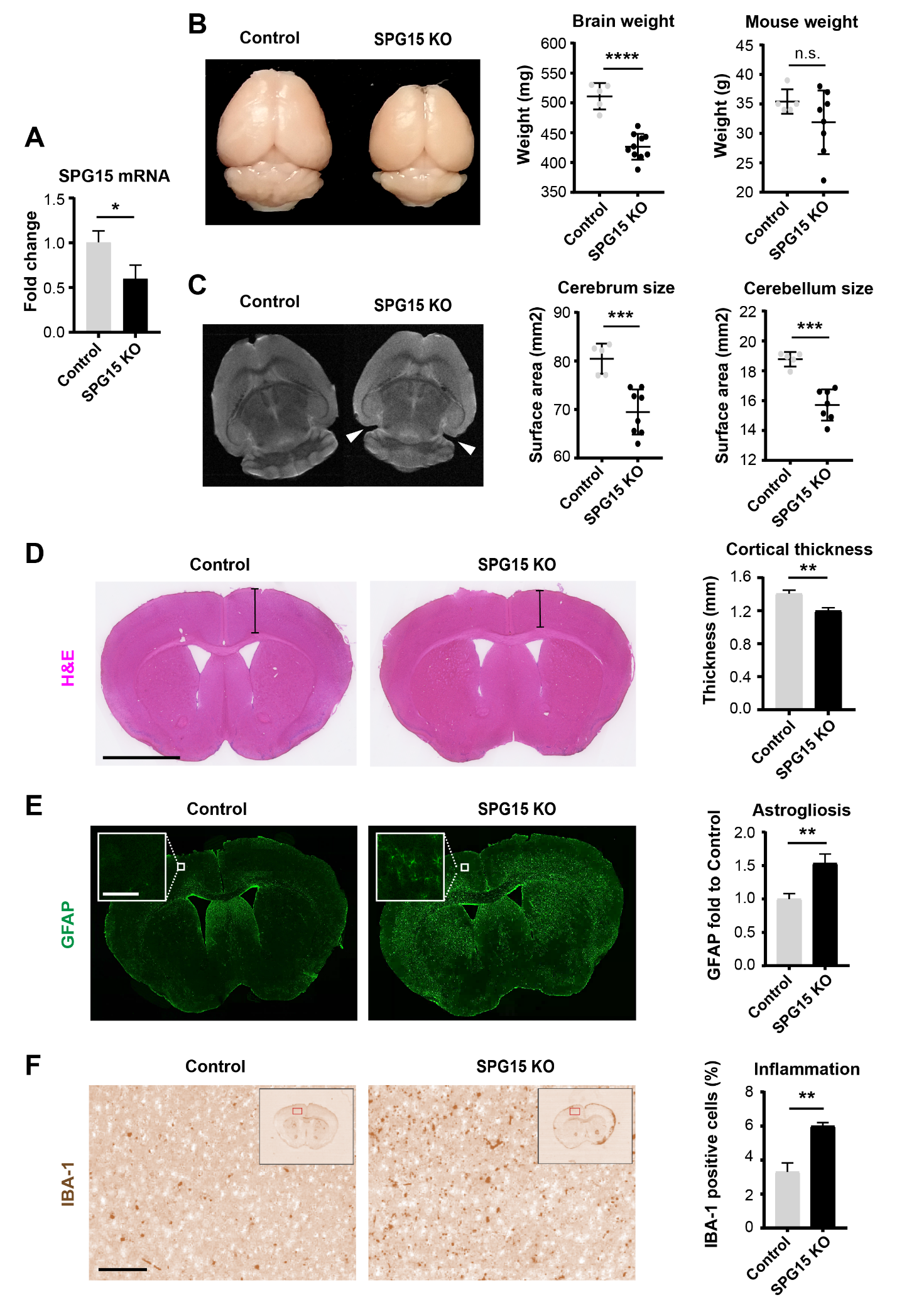


**Supplementary Figure 2. Brain pathology in SPG15 KO mice. Related to Figure 2.** A) Confirmation of SPG15 transcript reduction in SPG15 KO mice. N = 3, Unpaired t-test. Error bars represent SD. * corresponds to p<0.05. B) SPG15 KO mice exhibit visibly smaller brains irrespective of their overall body size, which is confirmed by quantification. Each data point represents an individual animal. t-test. **** corresponds to p < 0.0001. C) MRI axial slices of sample control and SPG15 KO mouse brains analyzed *ex vivo* (left). Quantification of cerebrum and cerebellum area from MRI sections (right) reveals a reduction in size of both anatomical structures in SPG15 KO mice, leading to the appearance of an interspace (white arrowhead) in diseased mice. Each data point represents an individual animal. t-test. *** corresponds to p < 0.001. D) Coronal sections stained with hematoxylin/eosin (H&E) show reduced cortical thickness in SPG15 KO mice. N = 3. t-test. Error bars represent SD. ** corresponds to p<0.01. Scale bar = 2.5 mm. E) GFAP immunofluorescent staining reveals diffused astrogliosis in SPG15 KO mice. N = 3. t-test. Error bars represent SD. ** corresponds to p<0.01. Scale bar = 100 μm. F) IBA-1 diaminobenzidine staining shows increased microglia infiltration in SPG15 KO mice. N = 3. t-test. Error bars represent SD. ** corresponds to p<0.01. Scale bar = 100 μm.


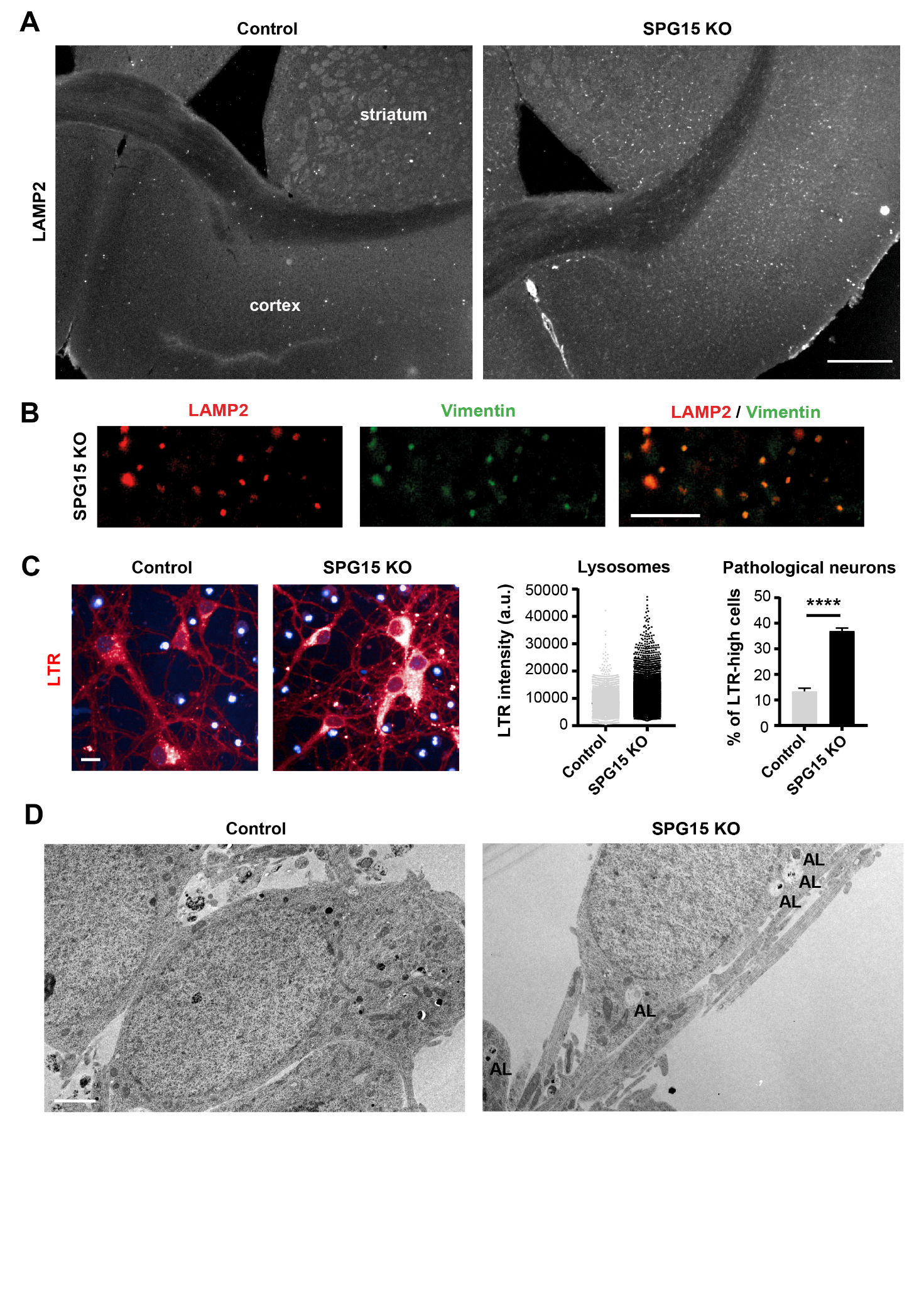
**Supplementary Figure 3. Characterization of lysosomes in SPG15 KO brain coronal sections and primary cortical neurons. Related to Figure 2.** A) Fluorescent micrographs showing LAMP2 stained sections of 15-month-old Control and SPG15 KO brains. LAMP2 signal is more evident in SPG15 KO slices due to lysosomes being aberrantly enlarged throughout the cortex. Interestingly, the striatum also shows lysosomal pathology. Scale bar = 500 µm B) SPG15 KO LAMP2 organelles often co-localize with Vimentin. Scale bar = 100 pixels. C) SPG15 primary cortical neurons show enhanced lysosomal staining as attested by increased cellular Lysotracker Red DND-99 (LTR) fluorescent intensity. Particularly, about 35% of neurons exhibit LTR values beyond the signal threshold deemed as normal. Scale bar = 10 µm. N = 3 (independent experiments, for a total of about 10,000 cells analyzed). Mann-Whitney test, error bars represent SEM. **** corresponds to p<0.0001. D) Transmission electron micrographs comparing control and SPG15 KO neurons. Contrary to controls, SPG15 KO neurons display numerous stalling autolysosomes (AL) filled with undigested material. Scale bar = 2 µm.


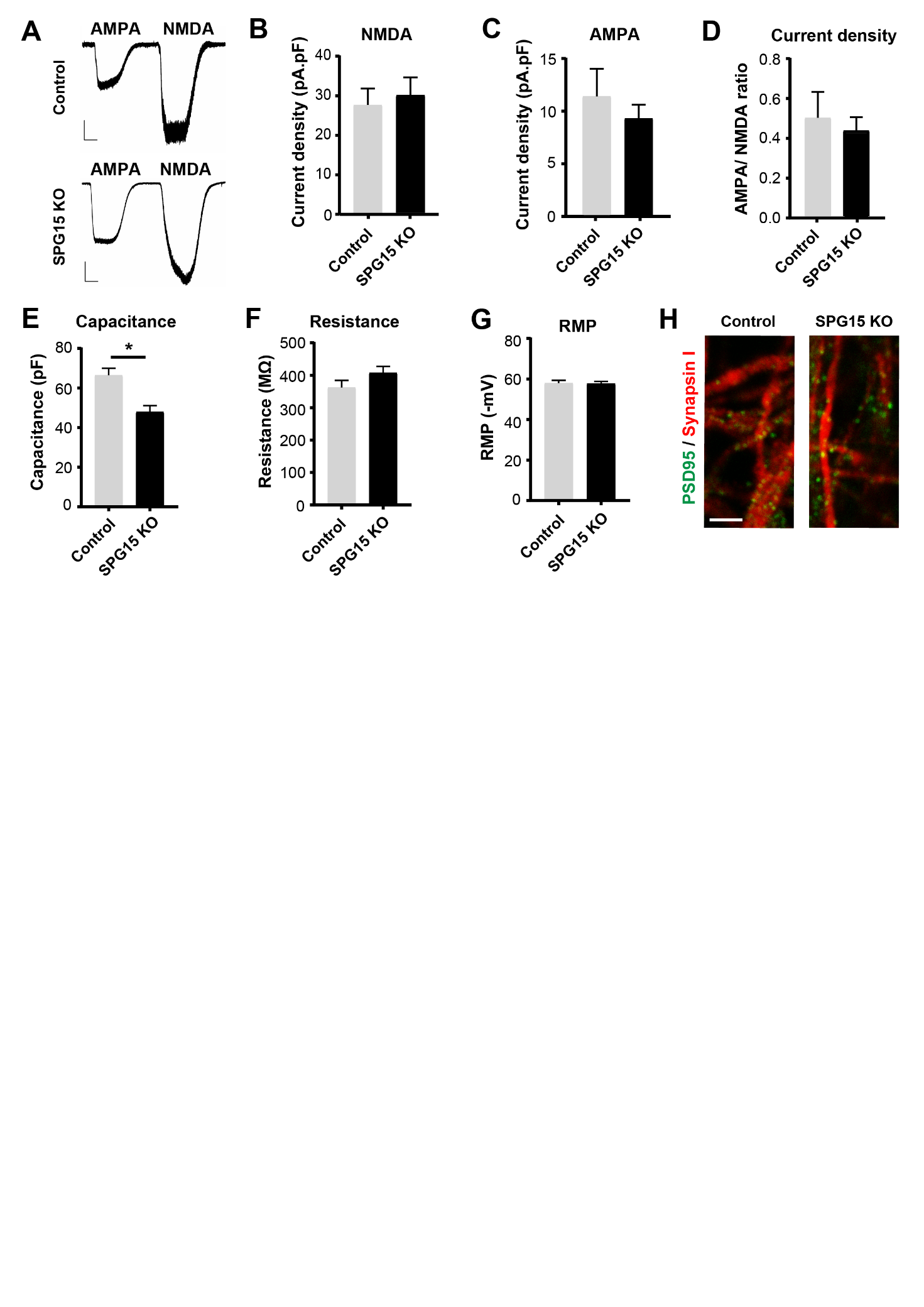
**Supplementary Figure 4. Electrophysiological characterization. Related to Figure 5.** A) Sample currents evoked by AMPA (100 µM) and NMDA (100 µM) in the presence of 50 µM glycine at a holding potential of -84 mV. Scale bar is 10 s, 200 pA. B) Mean ± SEM NMDA receptor current density (n = 21, N = 5). C) Mean ± SEM AMPA receptor current density (n = 21, N = 5). D) Mean ± SEM AMPA/NMDA receptor current density ratio (n = 21, N = 5). Intrinsic membrane properties of cells: whole cell capacitance (E), input resistance (F), resting membrane potential (G). H) Confocal micrographs highlighting synaptic contacts via Synapsin I (pre-synaptic marker) and PSD95 (post-synaptic marker) co-staining. N = 3. Scale bar = 2.5 µm.

**Supplementary Videos**

**Supplementary Video 1. Control fibroblasts with highlighted lysosomal tracks.** Time-lapse imaging of lysosomes from Control Fibroblasts stained with Lysotracker Red DND-99 at 9× normal speed. Acquired for 3 minutes, at 0.83 frames per second. Pink circles identify single lysosomes.

**Supplementary Video 2. Control fibroblasts.** Time-lapse imaging of lysosomes from Control Fibroblasts stained with Lysotracker Red DND-99 at 9× normal speed. Acquired for 3 minutes, at 0.83 frames per second.

**Supplementary Video 3. Patient fibroblasts with highlighted lysosomal tracks.** Time-lapse imaging of lysosomes from Patient Fibroblasts stained with Lysotracker Red DND-99 at 9× normal speed. Acquired for 3 minutes, at 0.83 frames per second. Pink circles identify single lysosomes.

**Supplementary Video 4. Patient fibroblasts.** Time-lapse imaging of lysosomes from Patient Fibroblasts stained with Lysotracker Red DND-99 at 9× normal speed. Acquired for 3 minutes, at 0.83 frames per second.

**Supplementary Video 5. Control cortical neurons and proximal lysosomal shuttling.** Control neurons are cultured in a microfluidic system that isolates axons from cell bodies. Time-lapse imaging of a proximal microchannel is shown at 5× normal speed. Lysosomes are stained with Lysotracker Red DND-99. Acquired for 2 minutes, at 5.8 frames per second.

**Supplementary Video 6. Control cortical neurons and distal lysosomal shuttling.** Control neurons are cultured in a microfluidic system that isolates axons from cell bodies. Time-lapse imaging of a distal microchannel is shown at 5× normal speed. Lysosomes are stained with Lysotracker Red DND-99. Acquired for 2 minutes, at 5.8 frames per second.

**Supplementary Video 7. SPG15 KO cortical neurons and proximal lysosomal shuttling.** SPG15 KO neurons are cultured in a microfluidic system that isolates axons from cell bodies. Time-lapse imaging of a proximal microchannel is shown at 5× normal speed. Lysosomes are stained with Lysotracker Red DND-99. Acquired for 2 minutes, at 2.6 frames per second.

**Supplementary Video 8. SPG15 KO cortical neurons and distal lysosomal shuttling.** SPG15 KO neurons are cultured in a microfluidic system that isolates axons from cell bodies. Time-lapse imaging of a distal microchannel is shown at 5× normal speed. Lysosomes are stained with Lysotracker Red DND-99. Acquired for 2 minutes, at 5.8 frames per second.

**Supplementary Video 9. SPG15 KO cortical neurons and lysosomal traffic jam.** Time-lapse imaging of a lysosomal traffic jam in SPG15 KO neurons is shown at 5× normal speed. Lysosomes are stained with Lysotracker Red DND-99. Only axonal terminals are considered in this view. Acquired for 2 minutes, at 2.6 frames per second.
